# Research progress on correlations between trace element levels and epilepsy

**DOI:** 10.3389/fcell.2023.1167626

**Published:** 2023-08-09

**Authors:** Wanyu Liu, Jingqing Xu, Linhai Zhang, Fangjing Li, Lijia Zhang, Zhenzhen Tai, Juan Yang, Haiqing Zhang, Jinmei Tuo, Changyin Yu, Zucai Xu

**Affiliations:** ^1^ Department of Neurology, Affiliated Hospital of Zunyi Medical University, Zunyi, China; ^2^ The Collaborative Innovation Center of Tissue Damage Repair and Regeneration Medicine of Zunyi Medical University, Zunyi, China; ^3^ Department of Nursing, Affiliated Hospital of Zunyi Medical University, Zunyi, China

**Keywords:** epilepsy, trace element, animal model, treatment, research progress

## Abstract

Research investigating the correlation between human trace element levels and disease alterations is growing. Epilepsy, a common nervous system disease, has also been found to be closely related to abnormal levels of trace elements. Studies continue to explore mechanisms of various trace elements involved in epileptic seizures through experimental animal models of epilepsy. Thus, we reviewed the research progress on the correlation between trace element levels and epilepsy in recent years and found that the trace elements most closely related to epilepsy are mainly metal ions such as selenium, iron, copper, zinc, and manganese. These results indicate that the changes in some trace elements are closely related to the increase in epilepsy susceptibility. In addition, after treatment with drugs and a ketogenic diet, the concentration of trace elements in the serum of patients with epilepsy changes. In other words, the abnormality of trace element concentrations is of great significance in the occurrence and development of epilepsy. This article is a literature update on the potential role of trace element imbalance in the development of epilepsy, providing new references for the subsequent prevention and treatment of epilepsy.

## 1 Introduction

Epilepsy is a prevalent neurological disorder characterized by persistent susceptibility to seizures, which can lead to various neurobiological, cognitive, psychological, and social ramifications. (Fisher et al., 2005; Fisher et al., 2014). An initial brain injury later results in neuron damage, ion pathway dysfunction, pine fibrosis, glial hyperplasia, synaptic plasticity, inflammatory response, and impairment of brain nerve function, typically resulting in the development of the pathological condition known as epilepsy. In order to prevent and treat epilepsy, it is therefore interesting and crucial to research whether the pathophysiology of epilepsy is related to changes in various trace elements in patients and animal models, leading to fluctuations in susceptibility to epilepsy. According to several pieces of research, the body’s antioxidant defense mechanism relies on the enzymes glutathione peroxidase (GSH-Px) and superoxide dismutase (SOD) to neutralize oxygen free radicals (Yüksel et al., 2000); furthermore, oxidative stress (OS) is the main mechanism underlying epilepsy in patients with epilepsy due to various causes (Zimmer et al., 2021). In particular, iron metabolism and ferroptosis are essential in epilepsy (Chen et al., 2020). Several enzymes, such as tyrosinase and dopamine hydroxylase, require copper as a cofactor. Under normal circumstances, the release of copper during synaptic transmission helps regulate neuronal excitability, while a decrease in serum copper levels can lead to epileptic discharge (Das et al., 2019). Furthermore, modulation of the Zn^2+^-metal-regulated transcription factor 1 (MTF1)-CaV3.2 cascade reaction also impacts epilepsy (van Loo et al., 2015). Notably, selenium is an antioxidant, and glutathione peroxidase (GSH-px), a selenium-dependent enzyme, reduces organic and hydrogen peroxides in the presence of reduced glutathione (GSH) (Nazıroğlu and Yürekli., 2013). Changes in blood manganese levels in epilepsy-susceptible rats can affect the activity of glutamine synthase (Carl et al., 1993). Moreover, treating epilepsy with drugs and a ketogenic diet (KD) resulted in changes in trace element levels. Thus, trace elements are crucial in the emergence of epilepsy.

Epilepsy has a significant impact on the lives and work of many patients. Despite receiving treatment with antiseizure medications (ASMs), approximately 30% of patients remain drug-resistant. At present, there are many treatment methods for epilepsy, such as the most commonly used drug therapy and surgical treatment; however, the efficacy of these treatments varies among individuals. Ongoing research explores interesting and potentially effective approaches to control epilepsy. Balanced trace element concentrations in people and animal models may aid in the treatment of epilepsy, according to certain clinical evidence or basic research findings. This article reviews and discusses the research on trace elements and epilepsy in recent years, the changes in the levels of trace elements in epilepsy, the possible mechanisms of trace elements involved in epilepsy occurrence, and how to improve epilepsy through these mechanisms.

## 2 Changes in trace element levels in patients with epilepsy

The interaction of trace elements with the human body has been the subject of extensive research over the last 30 years. During this period, people increasingly recognized the importance of these factors in human diseases, including their contribution to the global disease burden. In recent years, studying the relationship between epilepsy and trace element levels has become a new direction in anti-epilepsy regimens. Research has shown that the levels of micronutrients in children with epilepsy have changed, and further differences have been observed between drug-controlled epilepsy and refractory epilepsy (Verrotti et al., 2002; Ashrafi et al., 2007; Seven et al., 2013; Wojciak et al., 2013; Prasad et al., 2014; Saghazadeh et al., 2015; Dixit et al., 2020; Svetel et al., 2021). This article lists the changes in trace element levels in patients with epilepsy from multiple studies and analyzes the correlation of these levels with epilepsy.

### 2.1 Zinc

Zinc is essential for maintaining antioxidant activity, metabolic balance, and immune system function (Mocchegiani et al., 2008); furthermore, it has been widely studied because of its contribution to epileptic neurological function. The average serum zinc level in children with refractory epilepsy was found to be significantly lower than that in controls (95.8 ± 29 μg/dl *vs*. 145 ± 37 μg/dl; *p* < 0.001) (Saad et al., 2014). Another study similarly showed that the average zinc level in children with epilepsy is significantly lower than that in the controls (60.1 ± 22.6 μg/dl *vs*. 102.1 ± 18 μg/dl; *p* < 0.001) (Talat et al., 2015). Patients with epilepsy having serum zinc levels of <70 μg/dl exhibit a 3.56 times greater risk of epilepsy than do those having serum zinc levels of 70–120 μg/dl (Das et al., 2019). However, another study reported that the serum zinc concentration of untreated patients with epilepsy is significantly higher than that of controls (Saghazadeh et al., 2015), and there is no significant difference in zinc levels between the epilepsy and control group (Gündoğdu et al., 2023). In addition, a study on the treatment of refractory epilepsy in children using oral zinc supplementation showed that 31% of children in the intervention group exhibited improvement, while only 4.5% of children in the placebo group did, with a significantly lower frequency of seizures in the intervention group compared to the placebo group (*p* = 0.02) (Saad et al., 2015). Serum zinc concentrations in patients with epilepsy vary across different research findings. Hence, monitoring the serum zinc concentration of patients is necessary, and supplementing zinc during zinc deficiency may be an effective option during epilepsy treatment.

### 2.2 Iron

Hemosiderin, which is stored in the body as iron, is formed in the brain due to hemoglobin breakdown (Zhang et al., 2018). While epilepsy is intimately tied to inflammation, some cytokines (Sanz and Garcia-Gimeno., 2020), such as IL-6 and TNF-α, are associated with iron control and metabolism (Tombini et al., 2013). The presence of iron and its role in regulating inflammation within the epileptic brain can explain the impact of iron on the occurrence and development of epilepsy. One study reported that blood iron levels were substantially lower in the control than the epilepsy group (106 ± 8 μg/dl *vs*. 88 ± 8 μg/dl; p<0.01) (Ikeda, 2001). Additionally, some studies have discovered that certain seizure types might cause an increase in iron buildup in pyknotic neurons (Zimmer et al., 2021). Further, individuals with numerous metastases may experience iron overload due to medullary aplasias, hemolytic anemias, or hemochromatosis. Therefore, it is important to note that iron excess may be a factor in the second effect, the propensity to produce epilepsy by means of hypoxic damage, OS, and inflammation. Hence, regulating iron metabolism in the brain may provide a new strategy for epilepsy treatment.

### 2.3 Copper

Copper can indirectly enhance neurotoxicity by producing oxygen free radicals (Talat et al., 2015) and enhance neural excitability; furthermore, it is closely related to epilepsy. Copper alone inhibits magnesium and sodium-potassium ATPases, leading to changes in sodium and potassium levels, which in turn increases susceptibility to seizures (Donaldson et al., 1971; Garg et al., 2021; Nemati et al., 2021; Sharma et al., 2021). Some studies have shown an increase in serum copper levels in the epilepsy group compared to those of the control group (Wojciak et al., 2013; Gündoğdu et al., 2023), while others have reported no significant difference between the two groups (Sözüer et al., 1995; Chwiej et al., 2008). Another study found that the average copper level is significantly higher in the epilepsy group than in the control group (180.1 ± 32.4 μg/dl vs. 114.5 ± 18.5 μg/dl; *p* < 0.001) (Talat et al., 2015). Based on the above research results, we believe that controlling serum copper levels may improve the occurrence of epilepsy.

### 2.4 Selenium

GSH-Px, an antioxidant protein involved in glutamate-mediated excitotoxicity, membrane lipid peroxidation, neurotransmitter turnover, and implicated in the etiology of epilepsy, relies on selenium for its synthesis and functionality (Steinbrenner and Sies., 2013; Murthy et al., 2020; Abokrysha et al., 2021; Kamate et al., 2021). According to research, following rigorous screening, the average blood selenium level of epilepsy patients was 68.88 ± 17.58 ng/ml, whereas the control group exhibited a significantly higher average of 85.93 ± 13.93 ng/ml (*p* < 0.05) (Ashrafi et al., 2007). The study by Saad et al. (2014) demonstrated that the average serum selenium level of patients with epilepsy is significantly lower than that in the control group (4.2 ± 1.3 μg/dl vs. 8.3 ± 2.3 μg/dl; *p* < 0.001), which is consistent with the findings of Chakrabarty et al. (2022) (89.7 ± 13.6 μg/dl vs. 95.79 ± 12.66 μg/dl; *p* = 0.008). In contrast, Gündoğdu et al. (2023) reported that the average level of selenium was significantly higher in the epilepsy group than in the control group (71.95 ± 21.19 μg/l vs. 36.84 ± 9.08 μg/l; *p* < 0.001). Moreover, another study demonstrated that the serum selenium concentration did differ significantly between untreated patients with epilepsy and individuals in the control group (Das et al., 2019).

In summary, different clinical studies have shown conflicting changes in the levels of trace elements in the serum of patients with epilepsy; this may be due to factors such as insufficient sample size, individual differences, or differences in the environment or drug treatment. Some trace elements with antioxidant effects, such as zinc and selenium, may participate in and regulate some signal pathways and inhibit the occurrence of epilepsy when a stable concentration is maintained. In contrast, copper, iron, and other trace elements can increase the production of oxygen free radicals, enhance neurotoxicity, cause neural necrosis, and potentially increase susceptibility to epilepsy. Therefore, it is essential to monitor the concentration of various trace elements related to epilepsy in the serum for a long time during the anti-epilepsy regimen. If necessary, zinc, selenium, or other trace element supplement should be administered to maintain an adequate concentration of these elements and reduce epilepsy susceptibility. Simultaneously, the concentration of trace elements such as copper and iron that promote epilepsy should be regulated. These bidirectional control conditions may play a positive role in treating epilepsy. To obtain evidence of the correlation between trace element levels and epilepsy, more experimental data and animal model experiments are necessary. These studies would help support the influence of regulating trace element levels on the occurrence of epilepsy and to find countermeasures to reduce the occurrence of epilepsy.

## 3 Relationship between trace element levels and epilepsy in experimental animal models

The research on the changes in serum trace element concentrations in patients with epilepsy primarily focuses on quantifying these changes. Clinical studies on the mechanism of action of the trace elements in epilepsy are limited, while more exploration is conducted in experimental animal models.

### 3.1 Zinc and epilepsy

Zinc can be used as an anticonvulsant while homeostasis is maintained in the body and as a stimulant when homeostasis is disrupted (Prasad et al., 2014). Zinc homeostasis disorders are important in neurodegenerative brain diseases (Pavlica and Gebhardt., 2010). The Zn^2+^-MTF1-CaV3.2 cascade reaction, which occurs when high amounts of Zn^2+^ activate MTF-1, which subsequently binds to MRE in the CaV3.2 gene promoter and stimulates transcription of this gene, was discovered in a mouse pilocarpine epilepsy model (van Loo et al., 2015). The expression of CaV3.2 channels is notably increased, leading to elevated I_CaT_ levels due to the upregulation of CaV3.2 mRNA. Furthermore, the hippocampus network becomes more excitable as a result of the CA1 pyramidal cells’ conversion from normal discharge to burst discharge when I_CaT_ is elevated (Sanabria et al., 2001; Yaari et al., 2007). Thus, controlling this cascade reaction to reduce the frequency of recurrent epilepsy is an effective method (van Loo et al., 2015). A low zinc diet in epileptic mice can reduce brain zinc levels and increase susceptibility to epilepsy (Fukahori and Itoh., 1990), whereas a high zinc diet has the opposite effect. Notably, zinc affects brain function during seizures; if its concentration exceeds a certain level (>100 mM), it may cause the death of neural cells (Choi and Koh., 1998). Moreover, zinc can selectively inhibit N-methyl-D-aspartic acid (NMDA) and γ-aminobutyric acid (GABA) receptors, as well as glutamate and GABA transporters, to affect the occurrence of epilepsy (Pavlica and Gebhardt., 2010; Nazıroğlu and Yürekli., 2013; Carver et al., 2016; Das et al., 2019). Zinc can also influence the removal of free radicals since it is a component of the SOD, the most significant antioxidant enzyme (Horning et al., 2000; Yüksel et al., 2000; da Cunha et al., 2003). In summary, low or high levels of zinc in the brain can increase the susceptibility to epilepsy, and zinc seems to have an anti-epileptic effect when its homeostasis is maintained. Therefore, inhibiting zinc release, blocking channels, and modifying zinc transport and buffering in target tissues might all be useful regulatory strategies for treating epilepsy. Additionally, a workable strategy is to moderately supplement with zinc to maintain a balanced zinc level when there is a zinc deficit.

### 3.2 Iron and epilepsy

Numerous redox processes use iron molecules, such as DNA biosynthesis, tricarboxylic acid cycle, oxygen transport, and cellular oxidative respiratory chain. Although iron plays a crucial role in certain physiological processes, for the nervous system, iron accumulation caused by iron prolapse may increase susceptibility to epilepsy. Ferroptosis is a newly discovered form of cell death regulation, attributed to severe lipid peroxidation caused by reactive oxygen species (ROS) and iron overload as a result of multiple biological pathways involving ROS, redox GSH, glutathione peroxidase 4 (GPX4), intracellular iron ions (Fe^3+^ and Fe^2+^), and lipid peroxidation. In the process of iron ion interaction, the Fenton reaction produces ROS, which is the process of Fe^2+^ reacting with H_2_O_2_ to produce hydroxide and hydroxyl (Mittler, 2017). In addition, there is also a Fenton reaction process in the Haber Weiss cycle, which can generate ROS (Capelletti et al., 2020). ROS are produced more effectively by Fe^2+^, which also promotes lipid peroxidation and causes ferroptosis (Chen et al., 2020). Long-lasting convulsions in epileptic seizures encourage the generation of too much ROS, which causes oxidative stress and is intimately linked to the development of epileptic activity and neuronal cell death (Freitas, 2009; Eastman et al., 2020). Furthermore, increased ROS levels cause lipid peroxidation, which then results in iron deposition. A significant quantity of Fe^2+^ from hemoglobin and red blood cells is released into the brain in post-hemorrhagic stroke epilepsy (PHE) and post-traumatic epilepsy (PTE), which may cause ferroptosis (Mori et al., 2004). Ferroptosis can be less frequently seen, and epilepsy development can be delayed with reasonable regulation of ROS levels in the brain.

Additionally, glutamate (Glu), cysteine (Cys), and glycine (Gly) are the key components of the small molecule peptide GSH, which is essential for the body’s ability to eliminate ROS (Xiao and Meierhofer., 2019). GSH serves as GPX4’s substrate, which lowers the frequency of iron poisoning (Ursini and Maiorino, 2020). In epilepsy, GSH levels decrease, and ROS levels increase. Therefore, ferroptosis may be prevented, which is good for treating epilepsy, by controlling the amount of GSH and minimizing harm to neural cells in the epileptic brain.

### 3.3 Manganese and epilepsy

Manganese is the foundation for the development and function of nerve cells and another fundamental component of many metabolic processes in the brain. The brain’s extracellular fluid may be penetrated by Mn^2+^ and Mn^3+^, which can then bind to transferrin released by oligodendrocytes. In a non-binding state, it can be absorbed by choroidal plexus cells and brain parenchyma. Subsequently, neurons obtain ferritin-binding manganese through receptor-mediated endocytosis (Takeda, 2004). Through a transport system akin to neurons, manganese may also enter astrocytes. Once ingested by cells, it will interact with glutamine synthetase (GS) in astrocytes and SOD in neurons (Takeda et al., 1998). The lower capacity of the brain to metabolize glutamate and ammonia may be connected to the reduction in glutamine synthase activity in the brain of epilepsy-prone mice. The buildup of these chemicals may result in a local rise in pH and a local decrease in GABA-dependent chloride ion compression, both of which favor the diffusion of excitement (Carl et al., 1993). This indicates that manganese participates in and inhibits the metabolic pathway of glutamine synthase activity, which may lead to an increase in neural excitability and promote the occurrence of epilepsy. Regulating this pathway may be a new therapeutic target for epilepsy.

### 3.4 Selenium and epilepsy

Selenium controls or suppresses epileptic seizures caused by excitatory drugs (Nazıroğlu et al., 2008). In particular, the selenium-dependent enzyme GSH-Px is activated by selenium, and its activation allows GSH-Px to control the intracellular amount of hydrogen peroxide and hydroxyl radicals, hence controlling the antioxidant defense mechanism (Naziroğlu et al., 2009). In addition, 11 of the 15 neurodevelopmental disorders related to seizures can be caused by reduced expression of selenoprotein, low selenium status, interference of sodium selenate biosynthesis, or interruption of selenium transport into the brain through sodium selenate (Schweizer et al., 2021). In known animal studies, selenium can prevent the oxidation of nerve cell bodies and actively regulate brain function (Dominiak et al., 2016). However, high doses of selenium cause OS by consuming GSH, which reduces cholinergic signaling pathways’ activation and causes cholinergic neuron degeneration (Lakshmi et al., 2015). Thus, maintaining a selenium balance is important for epilepsy, as it can inhibit neuronal cell death through antioxidant effects and control epilepsy.

### 3.5 Copper and epilepsy

Copper is a cofactor of different enzymes. It is a component of copper-zinc-superoxide dismutase (Cu-Zn-SOD) and facilitates a neutralization in the accumulation of free radicals (Puig and Thiele., 2002). Notably, Cu-Zn-SOD not only has anti-epileptic effects by inhibiting cell apoptosis and altering the levels of Nav1.2/NRF2/HO-1 signaling pathway proteins in pilocarpine hydrochloride-induced epileptic rats but also significantly increases the postsynaptic potential latency in epileptic rats (Wen et al., 2022). Nevertheless, Cu’s capacity to accelerate redox processes might mistakenly result in the generation of ROS (Denoyer et al., 2015). In addition, significant brain inflammation brought on by copper is thought to have a role in developing epilepsy (Sozuer et al., 1995; Yilmaz et al., 2009; Płonka-Półtorak et al., 2013). Despite the antioxidant properties of Cu-Zn-SOD, the mechanism of the Cu-catalyzed redox reaction may raise the level of ROS, causing pro-inflammatory responses and raising the risk of neurotoxicity and epilepsy.

In summary, studies on experimental animal models have paid more attention to studying the complex mechanisms of trace elements involved in epilepsy rather than simply measuring the changes in trace element concentrations after epilepsy, which is conducive to discovering the role of various trace elements in epilepsy. Manganese may affect some metabolic and other pathways, reduce the activity of certain enzymes, and promote the occurrence of epilepsy. Further, zinc and selenium can reduce seizures and epilepsy susceptibility through antioxidant, neuroexcitatory, and neuroprotective effects. Although copper is an important component of Cu-Zn-SOD, which scavenges oxygen free radicals, copper itself can increase the erosion of oxygen free radicals on nerve cells, leading to cell death, increasing nerve excitability, and increasing epilepsy susceptibility. Excessive iron accumulation from iron deposition is also recognized as a contributing factor to epilepsy. Furthermore, various trace elements play different roles in epilepsy, and it is possible to determine the mechanisms by which some trace elements damage or protect nerve cells. From the perspective of epilepsy treatment, maintaining a balance of trace element levels in the body may effectively control the frequency and severity of epilepsy. After obtaining more accurate and reliable experimental data in the future, intervening in the pathways associated with these trace elements involved in epilepsy could emerge a novel and effective approach to treating epilepsy.

## 4 Effects of different anti-epileptic regimens on trace elements

### 4.1 Effects of drug therapy on trace elements

ASM treatment is the most widely used anti-epileptic regimen, and valproic acid (VPA) and carbamazepine (CBZ) are safe and effective ASMs. Studies from recent years claim that VPA medication can lower serum zinc levels (Graf et al., 1998; Armutcu et al., 2004; Płonka-Półtorak et al., 2011); furthermore, zinc levels have been shown to increase after VPA treatment (Altunbaşak et al., 1997; Hamed et al., 2004). Notably, after receiving VPA, individuals with epilepsy had considerably lower blood copper levels than did the control group (Kaji et al., 1992; Kuzuya et al., 1993; Liu et al., 1997; Armutcu et al., 2004). Specifically, VPA can promote the clearance of copper, selenium, and zinc, reducing the synthesis of free radical scavenging enzymes (Kürekçi et al., 1995). According to other research, there were no variations in the plasma concentrations of copper, zinc, or manganese among individuals receiving sodium VPA or CBZ monotherapy (Gonzalez-Reyes et al., 2007). Some studies also suggest that VPA and CBZ affect the intracellular distribution of zinc (Ilhan et al., 1999).

ASM treatment may affect the homeostasis of trace metal levels in the serum rather than affecting the seizure itself. Furthermore, trace element levels in the body will change while receiving ASM medication, which is difficult to prevent. Nevertheless, through ASM treatment, we can solely regulate the levels of trace elements that contribute to epilepsy, sustain the concentrations of trace elements beneficial for epilepsy, and manage the levels of trace elements that heighten susceptibility to epilepsy. Thus, it is crucial to gather more information through experimental animal studies to further elucidate the mechanisms underlying trace element concentration changes during ASM treatment. This will shed light on the effects of these changes and further enhance the safety of drug therapy for epilepsy.

### 4.2 Ketogenic diet

A highly specialized nutritional diet known as KD is distinguished by its proportional mixing of lipid and non-lipid (protein and carbohydrate) elements. Status epilepticus (SE) may impair intestinal food intake, affecting certain trace elements’ intake and increasing susceptibility to epilepsy. Furthermore, if an individual cannot tolerate a KD, parenteral nutrition can be administered to supplement the necessary trace elements (Dressler et al., 2018). Multiple studies have shown that KD positively affects the treatment of epilepsy, especially refractory epilepsy (Maalouf et al., 2009; Garcia-Penas, 2018; Ułamek-Kozioł et al., 2019). KD may alleviate temporal lobe hippocampal neurodegeneration in rats with epilepsy by activating acid-sensing ion channel 1a (ASIC1a) and mitochondrial-mediated apoptosis pathway (Qiao et al., 2022). While KD is relatively safe for treating epilepsy, some minerals, vitamins, and trace elements, including selenium (Lima et al., 2014; Arslan et al., 2017; El-Rashidy et al., 2020) and zinc (Christodoulides et al., 2012; Hayashi et al., 2013; Frommelt et al., 2014), can decrease in patients with epilepsy, making KD a double-edged sword. Due to the important role of zinc and selenium in the anti-epilepsy process, it is crucial to detect patients’ serum trace element levels during KD treatment and supplement them if necessary. Furthermore, studying the effects of combining KD with various anti-epileptic regimens is a promising area of research.

## 5 Discussion

**FIGURE 1:**
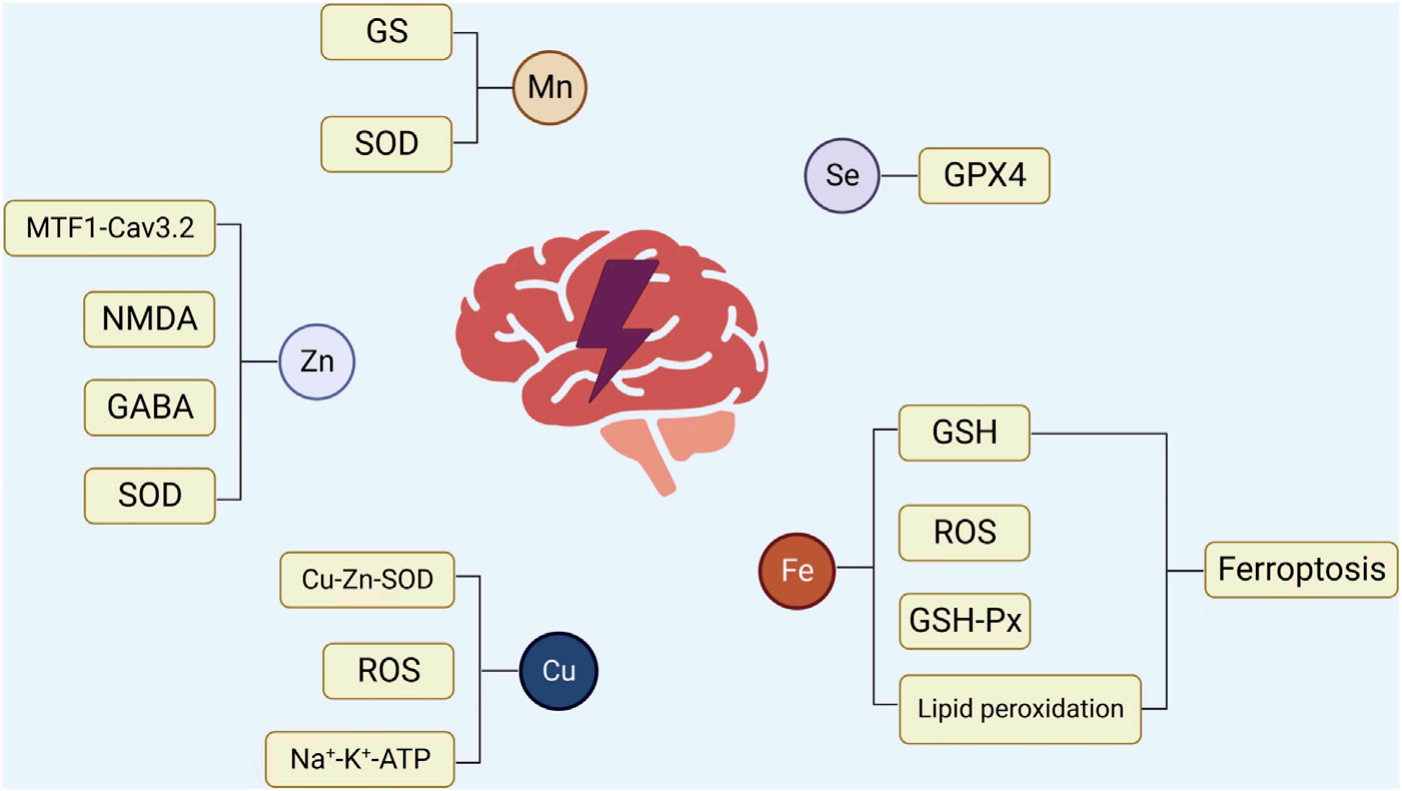
The relationship between epilepsy and trace elements. 1) Zinc is involved in regulating SOD, MTF1-CaV3.2 cascade reaction, NMDA, and GABA to affect the occurrence of epilepsy. 2) Selenium regulates the activity of GSH-Px, which contributes to glutamate-mediated excitotoxicity, membrane lipid peroxidation, and neurotransmitter conversion, thereby reducing the level of intracellular hydrogen oxide and hydroxyl radicalintracellular hydrogen oxide and hydroxyl radical levels. 3) Copper is involved in forming Cu-Zn-SOD, effectively prolonging the latency of epilepsy. However, copper can independently inhibit magnesium and sodium-potassium ATPase, and may produce OS in catalytic redox reactions, causing inflammation of nerve cells and promoting epilepsy. 4) Excessive accumulation of iron ions can produce a large amount of ROS, thereby influencing various biological pathways involving GSH, GPX4, GPX4, and lipid peroxidation. This heightened oxidative stress can subsequently increase vulnerability to epilepsy, affecting various biological pathways involving GSH, GPX4, GPX4, and lipid peroxidation and increasing susceptibility to epilepsy. 5) Manganese enters the nervous system in the form ofas ions, binds to SOD and GS in nerve cells, affects a series of metabolic pathways, and leads to the occurrence of epilepsy.

**FIGURE 2:**
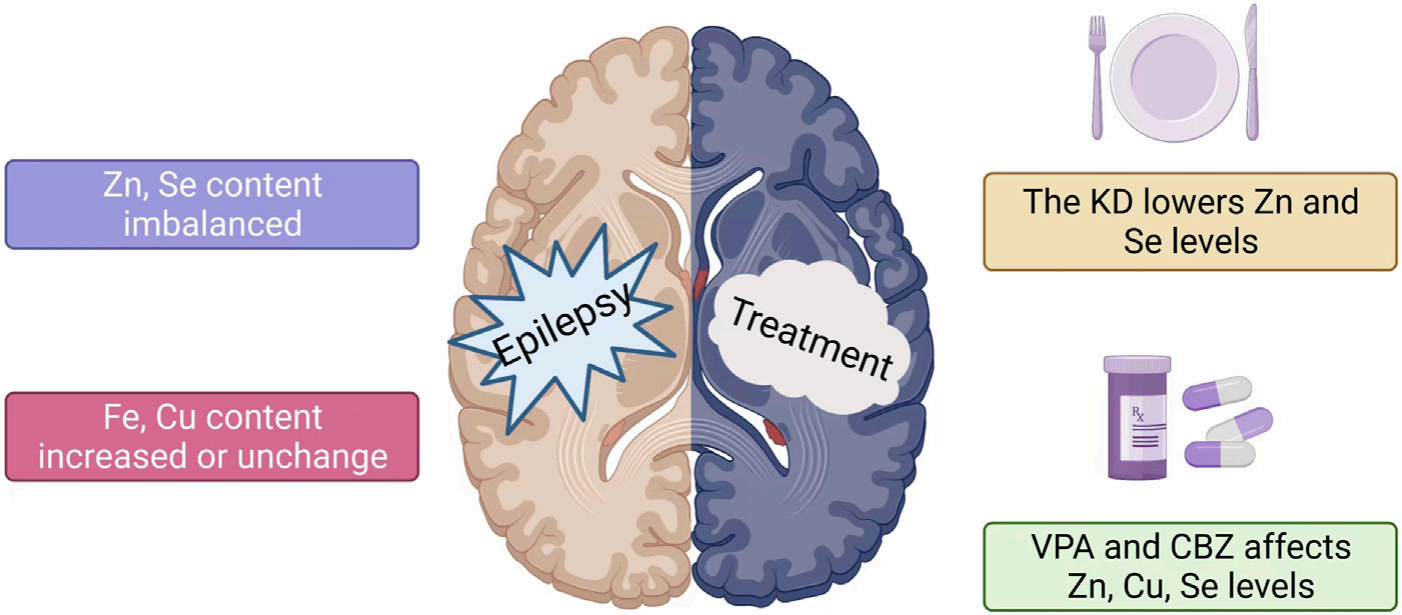
Changes in serum trace element levels in patients with epilepsy. The effect of different anti-epileptic methods on trace elements. 1) The trace element levels in patients with epilepsy vary, including those of zinc and selenium. Copper levels increase or remain unchanged, while iron levels increase. 2) KD reduces serum zinc and selenium levels in patients with epilepsy. 3) The changes in zinc and selenium levels in patients after VPA and CBZ treatment are not uniform.

In conclusion, the levels of trace elements in the patient’s body alter to various degrees following the beginning of epilepsy (Figure 2). Furthermore, the association between epilepsy and trace element levels is further evident in the participation of trace elements in regulating specific signaling pathways involved in epilepsy. For instance, zinc can affect the occurrence of epilepsy by participating in the regulation of pathways involving SOD, MTF1-CaV3.2 cascade reaction, NMDA, and GABA. Selenium is necessary for the synthesis and function of GSH-Px, which contributes to glutamate-mediated excitotoxicity, membrane lipid peroxidation, neurotransmitter turnover, and reduction in the levels of hydrogen oxide and hydroxyl radicals in cells. Although copper is an important component of Cu-Zn-SOD, which can prolong the incubation period of epilepsy, copper can independently inhibit magnesium and sodium-potassium ATPase and produce OS during catalytic redox reactions, causing nerve cell inflammation and promoting epilepsy. Elevated levels of intracellular iron ions (Fe^3+^ and Fe^2+^) will produce a large amount of ROS and affect various biological pathways, including those involving GSH, GPX4, and lipid peroxidation, leading to an increased susceptibility to epilepsy. Manganese can enter the brain as ions and combine with SOD and GS in nerve cells, thereby affecting metabolic pathways and leading to epilepsy. The above-mentioned trace elements play a role in the signaling pathways associated with epilepsy and therefore represent new therapeutic targets in treating epilepsy (Figure 1). In patients with epilepsy administered drug therapy, the levels of trace elements change for various reasons compared with those of the control group, and different ASMs may also be involved in these changes. Whether patients with epilepsy are newly diagnosed or have already undergone ASM treatment, long-term monitoring and timely adjustment of serum trace element levels positively affect controlling epilepsy. In addition, KD treatment can change the levels of trace elements in patients with epilepsy. Hence, during KD treatment, it is necessary to supplement trace elements such as zinc and selenium that can positively affect anti-epilepsy effects (Figure 2). Further research exploring the correlation between trace elements and epilepsy is required to understand the close relationship between *in vivo* trace element levels and epilepsy. This will contribute to a more comprehensive and effective anti-epilepsy regimen.
